# Weak Prezygotic Isolating Mechanisms in Threatened Caribbean *Acropora* Corals

**DOI:** 10.1371/journal.pone.0030486

**Published:** 2012-02-14

**Authors:** Nicole D. Fogarty, Steven V. Vollmer, Don R. Levitan

**Affiliations:** 1 Department of Biological Science, Florida State University, Tallahassee, Florida, United States of America; 2 Northeastern University, Marine Science Center, Nahant, Massachusetts, United States of America; Smithsonian's National Zoological Park, United States of America

## Abstract

The Caribbean corals, *Acropora palmata* and *A. cervicornis*, recently have undergone drastic declines primarily as a result of disease. Previous molecular studies have demonstrated that these species form a hybrid (*A. prolifera*) that varies in abundance throughout the range of the parental distribution. There is variable unidirectional introgression across loci and sites of *A. palmata* genes flowing into *A. cervicornis*. Here we examine the efficacy of prezygotic reproductive isolating mechanisms within these corals including spawning times and choice and no-choice fertilization crosses. We show that these species have subtly different mean but overlapping spawning times, suggesting that temporal isolation is likely not an effective barrier to hybridization. We found species-specific differences in gametic incompatibilities. *Acropora palmata* eggs were relatively resistant to hybridization, especially when conspecific sperm are available to outcompete heterospecific sperm. *Acropora cervicornis* eggs demonstrated no evidence for gametic incompatibility and no evidence of reduced viability after aging four hours. This asymmetry in compatibility matches previous genetic data on unidirectional introgression.

## Introduction

Botanists have long realized the importance of introgression (gene flow between species) as a key evolutionary process [1], [2], [3], [4]; however, only recently has the importance of introgressive hybridization been considered in animals [5], [6], [7], [8], [9], [10]. Outcomes of introgressive hybridization can range from the exchange of novel alleles to genetic swamping [8], [11], [12], [13]. When introgressed alleles are favored by selection, low rates of introgression may lead to adaptive shortcuts for the recipient species [2], [13], [14]. If there is sufficient selection against introgressed alleles, then ecological and morphological identity of the parental species will be maintained [13], [15]. Yet when introgression rates are high as a result of weak selection coupled with extensive hybridization, the loss of one or both parental species may occur via genetic swamping [16], [17]. Understanding the strength of selection and the reproductive isolating barriers will elucidate the evolutionary trajectory of hybridizing species and the likelihood of the above possible outcomes.

Sympatric broadcast spawning species must rely on temporal isolation (i.e., differences in time of gamete release) and gametic incompatibility (GI) to maintain species integrity [18], [19]. Gametic incompatibility is often defined as the reduced ability or failure of heterospecific sperm to fertilize eggs in the absence of sperm competition (no-choice crosses). However, GI also includes the preferential fertilization by conspecific sperm (i.e., conspecific sperm precedence –CSP) in sperm competition assays even if heterospecific gametes are compatible in no-choice crosses [20]. Testing for GI in competition magnifies the importance of fertilization rate (i.e., how quickly sperm can fuse with an egg in choice trials), as opposed to cumulative fertilization assays conducted without sperm competition (i.e., where sperm usually are given long intervals to find and fuse with eggs in no-choice crosses). Thus, to determine the hybridization potential between species, the likelihood of heterospecific fertilization in the absence and presence of conspecific sperm competition is crucial in determining the strength of gametic incompatibility.

Broadcast spawning corals tend to have high hybridization potential [5], [9]. Research on reproductive isolating barriers in scleractinian corals suggests that temporal differences in spawning times and gametic incompatibility can sometimes, but not always, be effective mechanisms of prezygotic reproductive isolation that prevent hybridization among congeners [21], [22], [23], [24], [25]. In *Acropora*, the most speciose coral genus in the world, at least 35 species have been observed to spawn in the Indo-Pacific within two hours of each other, many of which spawn synchronously [21], [26], [27]. No-choice laboratory crosses among these *Acropora* species showed varying degrees of hybridization potential with many species combinations showing some degree of heterospecific compatibility [28], [29], [30]. Choice trials were conducted between two compatible acroporid species pairs. Absolute conspecific sperm precedence where all of the larvae were sired by conspecific sperm [9] was demonstrated in 13 of the 14 crosses suggesting a strong prezygotic barrier to hybridization is present when sperm compete. In the Caribbean, *Montastraea annularis* and *M. faveolata* have overlapping spawning times but incompatible gametes. The third species, *M. franksi*, spawns an average of 100 minutes earlier than the species with which it is compatible, *M. annularis*, thus demonstrating strong temporal isolation [23], [25]. These temporal and gametic barriers appear to be very effective in some regions of the Caribbean, but in other regions the efficacy of the barriers are reduced and these species are genetically less distinctive [31].

The Caribbean acroporids, composed of *Acropora cervicornis* (staghorn coral), *A. palmata* (elkhorn coral) and *A. prolifera* (fused staghorn), is the only accepted naturally hybridizing coral system [9], [32], [33], but reproductive isolation in these corals has yet to be studied. *Acropora cervicornis* and *A. palmata* are sister species and are found in the fossil records dating back 6.6 [34] and 3.6–2.6 [35] million years, respectively; however *A. prolifera* has not been found in the fossil record [36]. Because of its historic rarity, its intermediate morphology, and marginal habitat preference, *A. prolifera* was suspected to be a hybrid of *A. palmata* and *A. cervicornis* [37], [38]. This was confirmed with molecular analysis which revealed all sampled *A. prolifera* colonies were heterozygous at three diagnostic loci, which is consistent with a first generation hybrid [32], [33]. Mitochondrial sequence data demonstrate that hybrids are produced from both *A. cervicornis* and *A. palmata* eggs [33]. Mitochondrial and nuclear data indicate that genes flow unidirectionally from *A. palmata* into *A. cervicornis* [32], [33]. For this one-way introgression to occur, hybrids must successfully backcross with *A. cervicornis*. Introgression rates varied across loci and showed a high degree of regional variation [33], [39], [40].

In recent decades the parental species have been reduced by 95% and are now listed as threatened species under the Endangered Species Act [41], [42], [43], [44], [45]. This decline is primarily attributed to a white-band disease outbreak in the 1980's [46], [47], [48], [49]. The hybrid has previously been documented as rare [9], [50], [51], [52], and it is unclear if they were susceptible to the disease outbreak. At some sites, hybrids can now be found at densities that exceed at least one of the parental species [53], [54]. Within the past 5 years, hybrid recruits have also been observed at Looe Key, Florida (S. Braynard pers. comm.), Sombrero Key, Florida (R. Ruzicka pers. comm.), and Curacao and Belize [55]. Across all studied life history stages (i.e., larval, settlement, early post-settlement, and adult), putative F1 hybrids were found to be as viable as the parental species [54].

Despite well characterized molecular evidence of hybridization and introgression, the reproductive isolation mechanisms that allow introgressive hybridization to occur and the conditions that may facilitate hybridization are unknown. In this study, we examine the strength of prezygotic mechanisms by using *in situ* field observations of spawning events to determine the extent of temporal isolation and a series of *in vitro* choice and no-choice fertilization crosses when gametes are fresh and when they have aged four hours.

## Methods

### Study taxa

The parental species have different morphologies which may dictate the habitat in which they live. *Acropora palmata* have robust elkhorn-like branches and often live in high wave energy environments. *Acropora cervicornis* has more finger-like branches and often live in calmer environments such as the back-reef or fore-reef areas [9], [37], [50], yet there are many sites throughout the Caribbean where the parental species overlap [50], [54], [55], [56]. The hybrid's morphology is intermediate to that of the parental species. Although it often lives in marginal nonparental habitats [9], [33], [57], it has recently been documented as growing next to, on top of, and interspersed with the parental species [55].

### Reproductive Biology of Caribbean Acroporids

There are a few studies that characterize the reproductive biology of *A. palmata* and *A. cervicornis* [58], [59], [60] but little is known about the hybrid. *Acropora palmata* and *A. cervicornis* are simultaneous hermaphrodites that reproduce once a year in late summer, July–September [58]. Each polyp releases an individual gamete bundle with spermatozoa packaged in the center surrounded by ova [60]. Prior to spawning the gamete bundle becomes visible (i.e., bundle setting) as it passes through the pharynx approximately 60–90 minutes prior to release (i.e., spawning). We compiled Caribbean-wide spawning observations from 1987-present on the coral-list server, previous publications [59], [60], [61], [62], [63], and our personal observations across the Caribbean and Florida. In addition, we compiled data from our 2004–2009 field efforts on the time acroporids were monitored, if spawning occurred, and the time gamete bundles were first observed and released (Table S1). In order to more closely scrutinize the potential for hybridization, we examined spawning times on nights when we observed both species spawning.

Most of our spawning monitoring efforts took place in Belize. Hybrids were difficult to monitor at this site because of the shallow, turbid environment in which they are found (<1 m). Because hybrids were monitored less frequently than the parental species, our limited hybrid spawning observations may not be a good indication of their reproductive potential. To increase the probability of capturing hybrid gametes, we haphazardly collected three to five hybrid colonies (20 cm in diameter) just prior to sunset and placed them in buckets on the dock.

### Study Sites

In 2004–2009 during the Caribbean acroporid spawning months (July–October), two sites typically were monitored for spawning throughout the Caribbean, the Florida Keys (lat: 24.545933, long: -81.40485), Belize (lat:16.80205, long: -88.08224), Panama (lat: 9.265, long: -82.12005), Curacao (lat: 12.08352, long: -68.89577), and Antigua (lat: 17.15794, long: -61.72992). Acroporid spawning was observed to spawn in the Florida Keys, Panama, and Belize. No-choice fertilization crosses (n = 20) between unique genets (originally genotyped by [59]) were conducted in the Florida Keys in 2004 and 2005. All other no-choice fertilization crosses (n = 88) and choice crosses (n = 9) were conducted in Carrie Bow Caye, Belize from 2005–2008.

### Microsatellite Genotyping

To reduce the likelihood of crossing clones mates (selfing) in fertilization assays, microsatellites were used to genotype the parental species in Belize prior to spawning in 2005 and to analyze the 2008 competitive crosses to determine paternity (see “Paternity Assignments” section below). In 2005, adult tissue samples were preserved in CHAOS (4 M guanidine thiocyanate, 0.1% N-lauroyl sarcosin sodium, 10 mM Tris pH 8, 0.1 M 2-mercaptoethanol) [31]. DNA extraction was conducted using methods described in [31] and genotyped using five microsatellite markers (loci 166, 181, 182, 187, and 201) and modified protocols of [59]. Each of the five microsatellite loci was PCR amplified separately as 20 µl reactions using Amplitaq (Applied Biosystems) according to manufacturers specification and a cycle (94°C for 2 min followed by 94°C, 30 s; 46°C, 30 s; 72°C, 45 s for 30 cycles followed by a final extension of 72°C for 3 min). Aliquots of all five PCR amplified loci were then mixed together (0.5 µl of each PCR product, 0.2 µl Liz standard, and 12 µl HD Formamide), run on a ABI 3100xl automated capillary sequencer with the Liz 500 size standard, and scored for size using Genescan v.3.1 and Genotyper v.3.7 software (Applied Biosystems). Within each sampled location, the probability that individuals share the same genotype by random chance and not by descent (i.e., identifying ramets as clonemates when they are not) was determined to be very low (p<0.0001)

### Field coral spawning observations and collection

Starting on the second day after the full moon in July–September, acroporid corals were monitored for spawning starting at 60–90 minutes after sunset. The number of divers varied with the site and year but ranged from two to six. Divers continuously monitored acroporid colonies for setting gamete bundles for approximately 90 to 180 minutes after sunset. If a colony was observed to be setting, the time was recorded and a net made of rip-stop nylon with a numbered collecting cup at its apex was secured over the colony. Once the buoyant gamete bundles were released, they floated to the top of the net where they were funneled into the removable cup. Divers recorded the species, spawn time, and collecting cup identity. Once a coral had finished spawning, a lid was secured to the collecting cup, and the cup was transported to the boat where the gamete bundles were concentrated. Most gamete bundles had not broken apart upon return to the laboratory (approx. 45 minutes after spawning); therefore, we gently swirled the cup to simulate wave action that would naturally occur in the field. Once gamete bundles dissipated, stock sperm and egg suspension was separated by pouring the suspension through a 120 µm Nitex filter. The eggs were retained on the filter and rinsed in filtered seawater four times to remove any sperm clinging to the egg's surface. More condensed, visibly cloudier sperm stocks were diluted to similar concentrations. One milliliter of all stock sperm suspensions were preserved and later quantified by conducting eight replicate sperm counts with a hemocytometer.

### Laboratory No-choice Fertilization Trials

No-choice fertilization trials were conducted 30 minutes after the gamete bundles broke apart by mixing 1 ml of sperm and 1 ml of eggs (approximately 100 eggs) of the same individual (self fertilization), of another individual of the same species (conspecific fertilization), or of a different species (heterospecific fertilization) in a 20 ml glass scintillation vial filled with eight ml of filtered seawater (0.45 µm). Control crosses were conducted by putting 1 ml of eggs into 9 ml of filtered seawater. Serial dilutions were conducted as per [23] to establish four to six 10-fold dilutions. After eggs and sperm were introduced, the scintillation vial was gently swirled three times and left undisturbed until fertilization was scored three to four hours later. Fertilization was determined by counting 50–100 embryos and unfertilized eggs. At this time, embryos were at least in the eight cell stage and easily distinguished from the round, smooth unfertilized eggs. In addition, gamete aging experiments were conducted to simulate what might occur in nature when adult densities are low and eggs may drift unfertilized for extended periods of time. No-choice crosses were conducted after both egg and sperm stocks had aged four to five hours. Since the same sperm stock solutions were used in these paired crosses (i.e., fresh gametes versus aged gametes), the sperm concentrations were equivalent. In Belize, the mean air temperature recorded above the open-air laboratory on nights across years when fertilization trials were conducted was 29.14°C (SE 0.03) (available for download at http://nmnhmp.riocean.com/arc_port.php) and was comparable to ambient sea temperature (28–30°C).

### Choice Fertilization Trials

Choice crosses (i.e., interspecific sperm competition) were conducted to examine the effectiveness of compatibility differences when sperm from both species are present. These crosses were conducted by adding 0.5 ml of *A. cervicornis* and *A. palmata's* sperm to 8 ml of filtered sea water, swirling the vial three times, and then adding 1 ml of *A. palmata* eggs. This experiment was then repeated with *A. cervicornis* eggs. In addition, two choice crosses for each species were conducted after all gametes had aged four hours to determine if gamete aging alters the outcome of competitive crosses. When possible adult tissue samples were collected from parent colonies and preserved, if that was not possible the remaining egg and sperm stocks were concentrated, preserved in twice their volume of CHAOS and stored at room temperature for molecular analysis to determine paternity. The larvae resulting from the choice crosses were reared for two to three days in order to have sufficient DNA for molecular analysis. Larvae were sacrificed by picking an individual larva with a Pasteur pipette and placing it to a 0.5 ml centrifuge tube containing 20 ul of CHAOS or 10% Chelex solution (BioRad) and stored at room temperature. Larvae from no-choice experiments were reared for five days and no significant difference in survival was observed between the three taxa [54]; therefore, it is unlikely that we added a postzygotic mechanism of hybrid larval inviability to our prezygotic study.

### Paternity assignments for choice trials

We determined paternity in two ways; in 2005, restriction digests of PCR amplification at MiniCollgen gene was used, and in 2008 we used allele identities at microsatellite markers. In 2005, larvae were genotyped using AluI restriction digest of PCR products from the MiniCollagen intron [33], [39]. A 373 bp fragment of MiniCollagen, containing the second intron of the gene, was amplified for each larva using published primers and protocols [33], [39]. The amplified PCR products were then digested using the restriction enzyme AluI (New England Biolabs), according the manufacturer's instructions, and then the restriction fragments were sized using super fine resolution 2% agarose gels (Amresco). Amplified MiniCollagen alleles for *A. palmata* and *A. cervicornis* contain three and four AluI cutsites, respectively, including a diagnostic AluI cutsite (AGCT) of the amplified PCR product for the *A. cervicornis* MiniCollagen alleles. This allowed us to identify larvae as hybrids or pure to either species based on the presence of species specific AluI restriction fragments, specifically a 149 bp band in *A. palmata* and a 122 bp band in *A. cervicornis*. Hybrid larvae were identified based on the presence of both the 149 bp *A. palmata* band and the 122 bp *A. cervicornis* band. Direct sequencing of undigested MiniCollagen PCR amplification from a subset of genotyped larvae (n = 32) confirmed that the restriction digest were 100% accurate.

In 2008, larvae and adults from choice crosses were genotyped using microsatellite protocols as described above (microsatellite genotyping) with slight modifications. DNA extractions were performed with a SprintPrep DNA Purification kit (Agencourt), a magnetic bead based protocol, and stored at −20°C until ready for use in PCR reactions. The PCR cocktail consisted of 2.8 µl double distilled water, 2.4 µl 5× PCR buffer (Promega), 1.5 µl 1.5 mM MgCl, 1.2 µl mM dNTPs, 0.15 µl GoTaq, 1.0 µl 10 µM bovine serum albumin, 0.5 µl of each primer pair, and 2.0 µl DNA (5 ng/µl). Contributing adults were run first to determine the most polymorphic loci for distinguishing paternity for each cross. At least two loci were used to confirm paternity using Genemapper software (Applied Biosystems, version 4.0). Only a few larvae (*A. cervicornis* = 9, *A. palmata* = 1) were observed to have more than two alleles at a locus.

### Statistics and analyses

Differences in spawning times were analyzed with a two-way ANOVA with species and night after the full moon as the fixed main effects. For each species, a one-way ANOVA was used to determine if spawning month influenced spawn time. The proportion of fertilized eggs for each species in no-choice crosses were analyzed using an ANCOVA with log transformed sperm concentration per milliliter as the covariate. Other variables (spawning year, location, and spawning month) were added to the ANCOVA model. The proportion of eggs fertilized were arcsine transformed for all no-choice crosses to meet the assumption of normality. A t-test was used to determine the difference in selfing rates between *A. palmata* and *A. cervicornis*. For each cross type a paired t-test was used to determine if there was a significant difference in fertilization when gametes were fresh (30 minutes after bundles had broken apart) and when they had aged four to five hour.

For choice crosses, other studies have used the success in no-choice crosses and relative sperm concentration to determine CSP [64], [65]. We did not make adjustments based on no-choice crosses because those adjustments are not appropriate when polyspermy (developmental failure caused by multiple sperm fusions) may be occurring. We used relative sperm concentration to determine the expected proportion of eggs fertilized based on the number of sperm collisions with an egg. For example, if a conspecific male had twice as many sperm as the heterospecific, the conspecific male would be expected to sire twice as many larvae because it has twice as many collisions. We generated a relative scale between −1 and 1 with positive values indicating conspecific sperm precedence and negative values indicating heterospecific sperm precedence. Significant deviation from a value of zero (no precedence) was determined using a chi-square test.

## Results

### Field spawning observations

**Figure 1:**
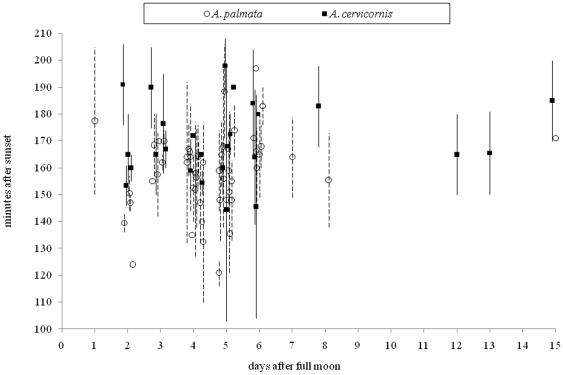
Spawning times for acroporids corals in Florida and the Caribbean. These data are taken from personal observations, publications, and postings on the coral-list server. Lines above and below the mean (symbols) indicate the given range of spawn times for *A. palmata* (dashed lines) and *A. cervicornis* (solid lines).

Unlike the coral *Montastraea* species complex that typically spawn on the same days after the full moon and at the same time after sunset each year [23], [25], Caribbean and Florida acroporids are less predictable in their spawning events. We only observed acroporid spawning on 14 of the 47 monitored nights (Table S1). Caribbean-wide spawning reports demonstrate that acroporids typically spawn two to six nights after the full moon and 120–200 minutes after sunset. Yet, there were observations as early as 1 night after the full moon and on nights around the new moon (Fig. 1). The average spawning times for *A. palmata* (159 min) and *A. cervicornis* (170 min) were significantly different (MS = 2218, df = 2, F = 5.82, p<0.01; SE *A. cervicornis* = 3.5 min and *A. palmata* = 3.3 min); however, there was much overlap in the spawning times of these two species (Fig. 1). Both species tended to spawn later after sunset on later evenings past the full moon, but the trend was not significant (MS = 3126.2, df = 10, F = 1.64, p = 0.11). There was no significant interaction between species and night after the full moon.

Caribbean-wide accounts suggest that the earliest date acroporids were observed to spawn was July 17^th^ and the latest was September 20^th^. The 79 Caribbean-wide spawning observations occurred over 6 days in July, 59 days in August, and 14 days in September. There was a significant month effect on spawning time (MS = 675.9, df = 2, F = 3.60, p = 0.04) for *A. palmata* but not *A. cervicornis* (p = 0,14). *Acropora palmata* colonies typically spawned earlier in August than July or September. Researchers in the Florida Keys are the only group to record a split-spawn (i.e., where spawning was observed during two consecutive months), but the rarity in split spawns is possibly a result of a lack of same site monitoring efforts over multiple months.

**Figure 2:**
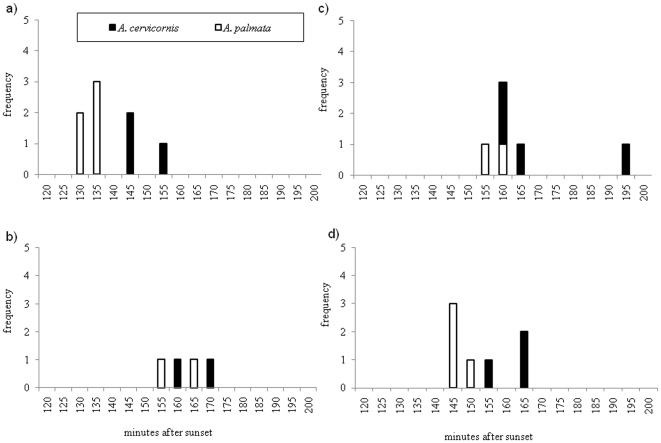
Observations of *A. cervicornis* and *A. palmata* spawning on the same night. We observed four separate spawning events where the parental species spawned on the same evening at: a) Florida Keys 2005, b) Belize 2005, c) Belize 2008, and d) Belize 2009.

Over a five year period, we monitored acroporid colonies on 47 nights from 2–6 days after the full moon (Table S1). Gamete bundle set 30–90 minutes prior to spawning. Acroporid colonies were observed to slowly release their gamete bundles over a 10–20 minute period. Both species spawned on the same night four times (Florida n = 1; Belize n = 3), only one species spawned on another four nights, and no spawning from either species was observed 25 times. It should be noted that a lack of spawning may be a result of colonies spawning the month prior to or after these monitoring efforts. On the four nights where both species spawned, the mean spawning times between species (*A. cervicornis* = 162 min and *A. palmata* = 147 min) were significantly different (ANOVA, MS = 632, df = 1, F = 6.96, p = 0.015; SE *A. cervicornis* = 2.9 and *A. palmata* = 3.0 min), but spawning times were abutting or overlapping on these nights (Fig. 2). On these four nights a significant difference in the night of spawning (ANOVA, MS = 1474, df = 2, F = 8.1, p = 0.002) was seen but there was no significant interaction between species and night after the full moon. Colonies in the Florida Keys (*A. palmata* = 136; *A. cervicornis* = 152) on average spawned earlier in the evening than colonies in Belize (*A. palmata* = 152; *A. cervicornis* = 167).

In 2006 one collected *A. prolifera* colony spawned in Belize within the typical range of parental species spawning times, 164 minutes after sunset on the fourth night after the full moon. Because no other acroporid coral spawned that night in the field, only self crosses could be conducted with this individual.

### Laboratory No-choice Conspecific and Heterospecific Crosses

Across sites and years, a total of 11 *A. cervicornis* genets and 21 *A. palmata* genets were used in no-choice crosses. Controls, where no sperm was added to unfertilized egg, were conducted for all crosses. Some controls were contaminated (i.e., eggs were fertilized), likely as a result of insufficient egg rinsing and self-sperm fertilization. If fertilization in the controls exceeded 10%, the corresponding crosses with the contaminated egg donor (*A. cervicornis*: n = 4; *A. palmata*: n = 2 crosses) were excluded from all analyses including aging and self crosses. The remaining controls used in this analysis averaged <3% fertilization (*A. cervicornis*: n = 11; *A. palmata*: n = 14).

**Figure 3:**
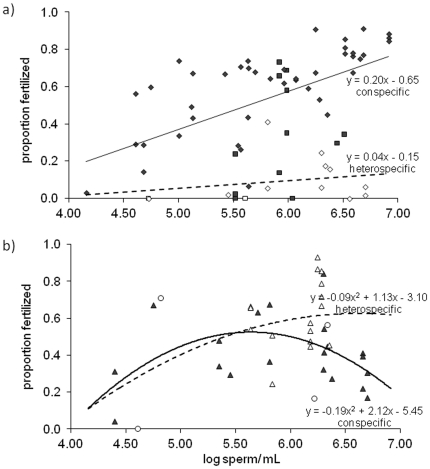
No-choice fertilization crosses demonstrating the proportion of eggs fertilized as a function of log sperm concentration. (A) Fertilization assays with *A. palmata* eggs where diamonds represent Belize crosses and squares Florida Keys crosses. (B) *A. cervicornis* eggs where triangles represent Belize crosses and circles represent Florida Keys crosses. Closed symbols and solid lines represent conspecific crosses and open symbols and dashed lines are heterospecific crosses.

In no-choice trials the pattern of fertilization differed between species, therefore each egg donor species was analyzed separately and tested against both species of sperm donors. No-choice crosses were conducted at two sites the Florida Keys (n = 12 conspecific; n = 7 heterospecific) and Belize (n = 62 conspecific; n = 26 heterospecific). Site had a significant effect on fertilization (p = 0.005). The average proportion of *A. palmata* eggs fertilized in the Florida Keys was significantly lower than crosses conducted in Belize. In Florida the average proportion of eggs fertilized in conspecific crosses (0.34) was much higher than heterospecific crosses (0.00), but because of low power this was not significant. There is a significant difference between the intercepts of the conspecific and hybrid crosses (p<0.0001) in Belize. We distinguish fertilization between the sites in Figure 3a, but pooled the sites together since the pattern of higher conspecific versus heterospecific fertilization was consistent at both sites. A total of 67 crosses with *A. palmata* eggs (n = 54 conspecific and n = 13 heterospecific) demonstrated a significant linear relationship between log sperm concentration and proportion of eggs fertilized (Table 1). These crosses needed more sperm to maximize fertilization compared to crosses with *A. cervicornis* eggs (Fig. 3a, Table 1). The interaction between sperm concentration and cross was not significant (p = 0.44). There was a significant difference in heterospecific and conspecific intercepts (Fig. 3a, Table 1) suggesting *A. palmata* eggs are much less compatible with heterospecific sperm than conspecific sperm.

**Table 1:** ANCOVA results of no-choice fertilization crosses.

species	source	df	type III SS	ms	*F*	*P*
*A. palmata eggs*						
	Log sperm (linear)	1	1.33	1.33	18.83	<0.0001
	Log sperm* log sperm (polynomial)	1	0.04	0.04	0.51	0.479
	Cross	1	3.84	3.84	54.56	<0.0001
	Cross*log sperm	1	0.42	0.42	0.59	0.440
	Cross*log sperm*log sperm	1	0.06	0.06	0.81	0.37
	Error	64	4.51	0.07		
	Total	66	9.37			
*A. cervicornis* eggs						
	Log sperm (linear)	1	0.29	0.29	5.06	0.031
	Log sperm* log sperm (polynomial)	1	0.27	0.27	4.67	0.037
	Cross	1	0.08	0.08	1.41	0.243
	Cross*log sperm	1	0.21	0.21	3.47	0.071
	Cross*log sperm*log sperm	1	0.2	0.2	3.7	0.062
	Error	36	2.08	0.06		
	Total	39	2.76			

ANCOVA was used to test differences in fertilization success as a function of cross type and sperm concentration. The dependent variable is the proportion of eggs fertilized (arcsine-transformed). The model consists of treatment group (conspecific vs. heterospecific cross) as the main effect, with sperm per milliliter (logistic transformation) as the covariate.

*Acropora cervicornis* eggs required an order of magnitude less sperm than *A. palmata* eggs to achieve fertilization. Only one *A. cervicornis* genotype spawned in the Florida Keys, therefore *A. cervicornis* conspecific crosses were only conducted in Belize. No significant difference (p = 0.14) in heterospecific fertilization with *A. cervicornis* eggs existed between the Florida Keys (n = 4) and Belize (n = 16), thus these two sites were pooled together. The 40 crosses were best fit by a polynomial factor (Table 1; Fig. 3b). A polynomial relationship in fertilization data is consistent with polyspermic fertilization, but eggs were not examined for multiple sperm infusion. Here, when eggs were scored for fertilization at least 3 hours after gamete introduction, fewer developed embryos were observed. The significant p-value of the polynomial and nonsignificant interaction provides evidence that both crosses with *A. cervicornis* eggs may experience polyspermy. There was no significant main effect of cross type (conspecific vs. heterospecific) and no significant interaction between sperm concentration and cross type (Table 1).

### Self fertilization

*Acropora cervicornis*, the species that requires less sperm to achieve fertilization, was also more susceptible to self fertilization. There was a significant difference (p = 0.046) in the average proportion of eggs fertilized by self sperm, with means of 0.04 (n = 6, SE = 0.03) and 0.21 (n = 8, SE = 0.06) in *A. palmata* and *A. cervicornis*, respectively. In 2006, self crosses were conducted in *A. prolifera* (hybrid) over varying sperm densities (1.3×10^2^–1.3×10^5^) and ages (30 minutes and 4 hours after gamete bundle dissipation). There was no self fertilization when gametes were fresh. After gametes had aged four hours, minimal fertilization (1–4%) was observed at moderate sperm concentrations (1.3×10^3^–1.3×10^5^), and no fertilization was seen at the highest sperm concentration.

### Gamete Aging

**Figure 4:**
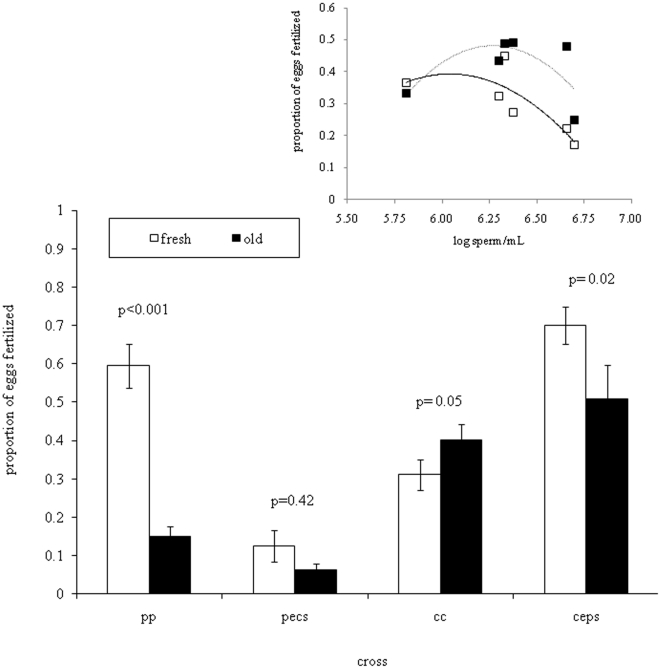
The effect of gamete aging on no-choice fertilization crosses. Open bars denote fresh gametes (30 minutes after bundle breakup) and shaded bars denote aged gametes (4–5 hours). Double letters represent conspecific crosses (p = *A. palmata* and c = *A. cervicornis*) and these letters followed by an “e” or “s” represent eggs or sperm for each heterospecific cross. Numbers above bars represent p-values and error bars represent standard error. The inlay represents the individual no-choice crosses for conspecific *A. cervicornis* fertilization trials, suggesting that a decrease in fertilization at high sperm concentrations (possibly a result of polyspermy) may be biasing the result of lower fertilization when gametes were fresh.

There was an effect of age on fertilization in all crosses except for hybrid crosses with *A. palmata* eggs (Fig. 4); however, the direction depends on the cross, mostly showing a decrease in fertilization with age. Conspecific crosses with *A. cervicornis* showed the opposite effect with increased fertilization as gametes aged (Fig. 4). Polyspermic fertilization provides a mechanism for this pattern; older sperm may be less likely to cause polyspermy resulting in higher fertilization. This is supported by the significant polynomial relationship of successful fertilization to sperm concentration in these crosses (ANCOVA, df = 1, F = 6.78, p = 0.04, Fig. 4 inset). There is no significant difference between these polynomials (p = 0.20) suggesting aging gametes four-five hours does not affect conspecific crosses with *A. cervicornis* eggs. Sperm concentrations were comparable, averaging 1.5×10^6^–2.8×10^6^ across cross types.

### Choice Fertilization Crosses

**Figure 5:**
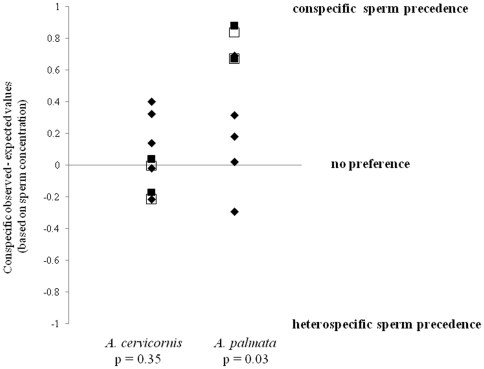
Choice crosses. Points denote the difference in the observed and the expected number of conspecific larvae sired for choice crosses. Expected values were calculated based on sperm concentration. Values of 1 would signify 100% of larvae sired by conspecific sperm, −1 would signify 100% of larvae sired by heterospecific sperm. Positive values denote conspecific sperm precedence (CSP), negative values represent heterospecific sperm precedence (HSP), and 0 represents no preference. Closed symbols represent competitive trials when gametes were fresh. Squares represent the four crosses where competitive trials were conducted when gametes were fresh (closed squares) and gametes had aged four hours (open squares).

The difference between the observed and expected number of conspecific larvae sired based on sperm concentration varied between species. There was no significant difference between the observed and expected number of conspecific larvae sired in *A. cervicornis'* (χ^2^ = 1.78, df = 6, n = 127, p = 0.72; Fig. 5); however, *A. palmata* eggs showed a significant difference (χ^2^ = 58.31, df = 6, n = 123, p<0.0001; Fig. 5), with most larvae being sired by conspecific sperm. There was no difference in the outcome of subset of competitive crosses (n = 2 for each species) where the gametes had aged four hours. For example, in crosses where CSP was seen when gametes were fresh, it was also seen when gametes had aged (Fig. 5).

## Discussion

### Prezygotic barriers

Our data demonstrated overlapping spawning times, a lack of gametic incompatibility in the eggs of *A. cervicornis*, and incomplete gametic incompatibility in the eggs of *A. palmata* all suggesting weak prezygotic barriers in Caribbean acroporids. Pooled spawning data from across the Caribbean demonstrated that although mean spawning times were statistically different, they showed a large degree of overlap. These data are crucial to understanding the window in which acroporids typically spawn, but it does not necessarily represent *A. palmata* and *A. cervicornis* colonies that spawned on the same night. Observations of sympatric parental species spawning on the same evening give more insight into temporal isolation between species. A previous coral study found that differences in *Montastraea spp.* spawning times (i.e., approximately one hour) may lead to temporal isolation [23]. Unlike a *Montastraea* colony that will release its gamete bundles in one synchronized pulse (N.D. Fogarty pers. obs.), an acroporid colony dribbles its gamete bundles over an extended period of time, making it more likely that gamete mixing of sympatric *A. palmata* and *A. cervicornis* colonies will occur. We observed that peak spawning times in sympatric *A. palmata* and *A. cervicornis* abutted (i.e., difference in spawning times of 5–10 minutes) or overlapped (i.e., spawn within 5 minutes of each other). While it might be slightly more likely for conspecifics to spawn simultaneously than heterospecifics, overlapping spawning times suggest temporal isolation is lacking in Caribbean acroporids.

This study found species-specific differences in gametic incompatibilities. The ease of fertilization in *A. cervicornis* eggs likely makes them susceptible to heterospecific, self, and possibly polyspermic fertilization and prolongs their viability by allowing fertilization to occur up to four hours after spawning. Although we did not examine *A. cervicornis* eggs for multiple sperm penetrations, the polynomial trend in the *A. cervicornis* fertilization data and the increase in fertilization with gamete age are consistent with polyspermy. After three hours, *A. cervicornis* sperm vary in their motility ranging from inactive to spastic (ND Fogarty pers. obs.). The loss of motility after sperm have aged, effectively lowers the sperm concentration reducing the probability of multiple sperm fusions, i.e., polyspermy. *Acropora palmata* eggs generally are more resistant to fertilization, needing an order of magnitude more sperm than *A. cervicornis* to maximize conspecific fertilization, have lower rates of heterospecific fertilization, and demonstrate reduced viability after four hours. This correlation between the difficulty in conspecific fertilization and lower heterospecific fertilization has also been found in other marine organisms such as sea urchins [66], and may explain why introgression of *A. cervicornis'* genes into *A. palmata* does not occur [33] or occurs at such low frequencies that a large sample size is needed for its detection [67].

Results of the choice assay mirror no-choice trials. *Acropora cervicornis* eggs show no evidence of discriminating against heterospecific sperm, while most *A. palmata* crosses demonstrated CSP. Because the CSP barrier was never absolute (i.e., conspecific males sired the majority, but not all of the larvae), occasionally heterospecific fertilization will occur in competition. The high variance in our choice crosses with *A. palmata* eggs is interesting, but not atypical. Variation in CSP among crosses has been found in other marine taxa (reviewed in [20], sea urchins [64], fish [65], sea stars [68]) and might reflect intraspecific variation in gametic compatibility often seen in a broadcast spawners [66], [69], [70], [71], [72], [73], [74]. The variation found in choice crosses in this study also may be attributed to the variation in sperm motility seen among *A. palmata* individuals (M. Hagedorn, unpub data). To our knowledge, we made the first attempt to examine the effects of gamete age in choice trials. Our study demonstrates that gamete aging often reduces fertilization in no-choice trials. Yet, the outcome of choice trials remained consistent over a four hour period suggesting that recognition mechanisms do not breakdown after a few hours.

It is not uncommon to find asymmetries in gametic incompatibility and introgression (cottonwoods [75]; fish [76]; birds [77], corals [29]; oak [78], [79]; sea urchins [66], mosquitoes [80], poplars [81]). Asymmetries in gametic incompatibility, found here, are consistent with previous genetic studies demonstrating unidirectional introgression with genes flowing from *A. palmata* into *A. cervicornis* [32], [33], [39]. In order for this unidirectional introgression to occur, *A. cervicornis* must repeatedly mate with the hybrid and form a backcross generation. Backcross individuals could be formed through *A. cervicornis* eggs being fertilized by hybrid sperm or hybrid eggs being fertilized by *A. cervicornis* sperm. Because the parental species did not spawn the night a hybrid spawned, we were not able to collect backcross data. We hypothesize that the promiscuity of *A. cervicornis* eggs may make them susceptible to fertilization by hybrid sperm. Because *A. cervicornis* sperm fertilized *A. palmata* eggs at lower rates, it is possible that the reciprocal backcross (i.e., *A. cervicornis* sperm fertilizes hybrid eggs) would occur at lower frequencies.

### Conclusions

Over the past decade we have gained a better understanding of the Caribbean acroporid system. Eggs of both parental species can form hybrids, albeit it is more likely with *A. cervicornis* eggs. Hybrids currently have a Caribbean-wide distribution, vary in abundances from being locally rare to exceeding the abundance of the parental species, are found overlapping with the parental species' habitat, and are equally as viable as the parental species [54]. In addition, new hybrids have been observed to recruit to the reef in recent years [55]. It has been suggested that Caribbean acroporid hybridization is either a relatively recent phenomenon or the environment has only recently favored hybrids [9]. The latter hypothesis would be more plausible if currently, hybrids were restricted to marginal, nonparental habitats. It is possible that hybrid formation is relatively more common recently than historically.

Prezygotic barriers are weak and there appears to be no postzygotic selection acting on F1 hybrids [54], yet hybrids are not found in the fossil record [36]. The mechanism preventing hybridization for millions of years might have been compromised recently. Density dependent prezygotic barriers may explain why hybridization is a recent phenomenon. Historically, when the parental species were highly abundant, eggs likely were swamped by sperm from neighboring conspecifics, limiting the number of hybrid embryos formed. With the recent decline in the parental species, sperm concentrations are lower and eggs are likely not fertilized immediately. Although it may take only minutes for *A. cervicornis* eggs to float away from spawning conspecifics and encounter heterospecific sperm, the prolonged longevity in *A. cervicornis* eggs further increases the probably of heterospecific fertilization. However, increased hybrid embryo formation from a reduction in the parental species populations can only occur to a certain point. If the parental species' densities are too low, the Allee Effect (i.e., the inability of eggs to be fertilized as a result of low sperm concentration) will prevent any fertilization [82].

Incomplete prezygotic barriers found here, may allow for variation in hybrid formation and perhaps subsequent introgression across sites. Previous studies demonstrate when ecological conditions change for hybridizing species, the balance of selection and introgression may shift, become unstable, and possibly lead to genetic swamping [17]. Endangered taxa are particularly vulnerable to genetic swamping; yet, these taxa are also at risk of inbreeding depression and may actually benefit from the acquisition of some genetic variation through introgression [14], [16], [17]. Although the evolutionary trajectory of Caribbean acroporids is unclear, what is obvious is that the survival of this genus will hinge on its ability to avoid extinction from the current onslaught of factors diminishing corals worldwide.

## Supporting Information

Table S1 Dates of spawning collection monitoring efforts. A summary of the dates and times when we have attempted to observe and collect acroporid spawn. If spawning did occur, the time gamete bundles were first observed in the mouth of the polyp was recorded as “bundle set time,” the range of spawning times was recorded below each species name, and the “bundle breakup” is the time in which the gamete bundles dissipated. “ns” represents that no spawning was observed, and “x” indicates that we did not monitor that species for spawning.
(XLS)
